# Understanding Rural Health through Rae Garringer’s Country Queers: A Love Letter

**DOI:** 10.13023/jah.0703.11

**Published:** 2025-09-01

**Authors:** Bradley A. Firchow

**Affiliations:** University of Kentucky College of Medicine

**Keywords:** Appalachia, cultural competency, health disparities, health equity, healthcare access, LGBTQ2+ health, sexual orientation, rural health, sexual and gender minorities, social determinants of health

## Abstract

Rae Garringer’s Country Queers: A Love Letter examines the experiences of queer individuals in rural America, emphasizing the relationship between place and identity. Through oral histories, the book presents perspectives on community, resilience, and healthcare access. While not centered on medical care, it highlights challenges in obtaining affirming services in rural areas. Garringer’s experience as a standardized patient provides additional context on healthcare gaps. For public health professionals, Country Queers offers insight into the structural and social factors affecting rural LGBTQ2+ populations, contributing to a broader understanding of community health and identity in Appalachia and similar regions.

Rae Garringer’s *Country Queers: A Love Letter* is rooted in the land, in the grit of human experience, and in the quiet determination of those who make home in places often dismissed as inhospitable. It is a collection of voices — a testimony to LGBTQ2+ people who have long shaped rural America’s backroads and hollers, yet whose stories are rarely told. This book does not simply document LGBTQ2+ life in rural spaces — it insists on it. For readers of the *Journal of Appalachian Health*, Garringer’s work challenges and expands prevailing ideas about health, community, and identity in a region often defined by its deficits rather than its complexity.

Launched in 2013 and shaped by a 7,000-mile solo road trip the following year, the *Country Queers* oral history project centers the lives of LGBTQ2+ individuals from 21 of the United States (U.S.), primarily in the Appalachian Region and the rural South. The book features more than 90 interviews with participants ranging in age from 19 to 81, most of whom are white, cisgender, and working-class. Its thematic organization around land, family, faith, and community reveals the entangled forces that shape rural LGBTQ2+ existence. Garringer’s consent-based, community-embedded approach affirms that those most affected by social conditions are the best narrators of their own lives.

While the book is not centered on healthcare, it contains rich, specific accounts of how medical care intersects with rural LGBTQ2+ life. Narratives touch on emergency medical situations, navigating HIV/AIDS treatment, and enduring care in contexts where providers often lack cultural humility. One participant recalls how AIDS devastated their social circles: “You’d see them one week, and they looked all healthy. Within a matter of months... they had AIDS. But you never really wanted to discuss it.” Silas House, Kentucky’s first openly gay poet laureate, shares how the epidemic shaped his understanding of self: “Besides the religion, also that consciousness and awareness of AIDS really held me back... I thought I would be dead to [my parents]. I thought I would lose my whole family.”

These stories show how rural health disparities are not just logistical but deeply emotional. Health and wellbeing are shaped not only by access to services but also by cultural attitudes, social ties, and the ability to live openly. As one narrator shared, their partner, Michael, who died of AIDS, “spent forty-five days in the hospital the first time,” but refused to take the prescribed medications. “His only strife in life... was that he wanted his family to give him some acceptance.” Another interviewee described using medical articles to help their parents reject conversion therapy: “My dad was just like, ‘Oh, okay.’” These moments reveal how health outcomes hinge on cultural belonging as much as clinical intervention.

Garringer’s background as a standardized patient in a rural medical school adds another layer of resonance. In a hospital gown between student exams, the idea for *Country Queers* emerged: “What if I could take a road trip to interview other rural queer people?” This moment, though quiet and mundane, captures a central theme of the book: the tension between what rural LGBTQ2+ people are told is possible and what they make possible for themselves.

For rural clinicians, health educators, and policymakers, *Country Queers* underscores the need to rethink rural health through the lens of identity, belonging, and place. It makes a case not for more cultural competency — an outdated model that suggests a finite body of knowledge — but for cultural humility: an ongoing, relational awareness that rural LGBTQ2+ people are not outsiders in their own communities. Particularly in Appalachia, where providers often share backgrounds with their patients, this shift requires recognizing both shared experience and difference.

The book’s implications are clear. Health systems must invest in training that affirms LGBTQ+ identities, support inclusive community spaces, and recruit providers with lived experience. Policy should reflect that rural LGBTQ2+ wellbeing cannot be separated from structural realities like geographic isolation, economic precarity, or lack of legal protections. For public health professionals, *Country Queers* offers a blueprint for understanding rural health in its full, intersectional dimensions.

This book is a love letter — but not a sentimental one. It is sharp-eyed and grounded. It asks us to recognize the realities of LGBTQ2+ life in our rural communities, to name the injustices still present, and to *truly listen* to voices that have always been here. *Country Queers* insists that rural LGBTQ2+ people are not anomalies. They are resilient, rooted in the soil, and very much at home.
